# Topical Brimonidine Delays Ultraviolet Radiation‐Induced Squamous Cell Carcinoma in Hairless Mice

**DOI:** 10.1111/php.13622

**Published:** 2022-04-13

**Authors:** Fernanda E. Pinto, Peter Olsen, Martin Glud, Hans Christian Wulf, Catharina M. Lerche

**Affiliations:** ^1^ 53146 Department of Dermatology Copenhagen University Hospital Copenhagen Denmark; ^2^ Department of Pharmacy University of Copenhagen Copenhagen Denmark

## Abstract

We investigated whether topical brimonidine delayed or enhanced the development of squamous cell carcinoma (SCC) when ultraviolet radiation (UVR) was applied to a well‐established murine model. Hairless female mice (*n* = 125) were randomized into five groups and treated as follows: 1% brimonidine cream before UVR (Group 1), 0.33% brimonidine gel before UVR (Group 2), 1% brimonidine cream after UVR (Group 3), UVR only (control; Group 4) and 1% brimonidine cream only (control; Group 5). For each animal, the first four tumors were recorded and followed until three tumors reached 4 mm or one tumor reached 12 mm in diameter. All animal experiments continued for up to 365 days or until death. Application of 1% brimonidine cream before UVR delayed tumor development relative to control mice treated with UVR alone (*P* = 0.000006). However, when 0.33% brimonidine gel was applied before UVR (*P* = 0.313) or 1% brimonidine cream was applied after UVR (*P* = 0.252), there was no significant delay in tumor development relative to control mice treated with UVR alone. The development of the second and third tumors followed a similar pattern. Topical 1% brimonidine cream applied before UVR exposure delayed SCC development in hairless mice. In contrast, when brimonidine was applied after UVR there was no significant delay in tumor development. These results suggest that the 1% brimonidine cream probably absorbed the UVR, and therefore, a delay in tumor formation was only seen when brimonidine was applied before irradiation. However, there can be multiple reasons for this delay in photocarcinogenesis.

## Graphical abstract

We investigated whether topical brimonidine delays the development of squamous cell carcinoma (SCC) induced by ultraviolet radiation (UVR) in hairless mice treated with brimonidine cream and after exposure to UVR. Animal tumors were recorded and followed until three tumors reached 4 mm or one tumor reached 12 mm in diameter. Animal experiments continued for up to 365 days or until death. Topical application of 1% brimonidine cream before UVR exposure delayed SCC development in hairless mice. Delay of SCC, induced by UVR in hairless mice, was probably due to UVR absorption by the drug.

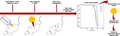

## INTRODUCTION

Brimonidine is a selective α_2A_‐adrenergic agonist (1) that is synthesized from a quinoxaline base (2) and exhibits diverse biological activities, such as neuroprotection (3), vasoconstriction (4) and anti‐inflammatory effects (4, 5). It is routinely used to lower intraocular pressure and has also been used to treat facial erythema in patients with rosacea (6) by reversing the vasodilation of superficial blood vessels in the skin (1, 7, 8, 9). Facial flushing, erythema and a burning sensation have all been described as adverse effects of the drug by patients with rosacea (1, 8, 9, 10).

Nizari *et al*. (3) confirmed the neuroprotective effect of brimonidine in different glaucoma‐related models and demonstrated that this effect is mediated by a soluble amyloid precursor protein. Piwnica *et al*. (4) used *in vitro* vascular and *ex vivo* human skin biopsy neuroinflammation models to show that brimonidine stimulated vasoconstriction of human subcutaneous arteries and vessels with diameters of <200 µm. Other studies have demonstrated that brimonidine has anti‐inflammatory properties (4, 5). These studies showed that brimonidine could reduce edema by up to 76%, compared to the vehicle (4), and could also reduce acute inflammation of the skin by promoting vasoconstriction and/or neutrophil migration (5).

Ultraviolet radiation (UVR) exposure is responsible for approximately 90% of keratinocyte skin cancers, which include squamous cell carcinomas (SCCs) and basal cell carcinomas (BCCs) (11). Bouvier *et al*. (12) investigated the anticarcinogenic potential of different concentrations of brimonidine (0.18%, 1% and 2%) on hairless mice exposed to UVR and showed that brimonidine delayed tumor development. Brimonidine was applied before UVR exposure on 3 days and after UVR exposure on a further 2 days per week for 1 year (12). There are many patients using brimonidine on UVR‐exposed areas for years. Therefore, this study aimed to investigate the time to tumor development in groups only receiving brimonidine before irradiation or after irradiation and not a mixture. This was done to clarify the photoprotective effect of brimonidine in combination with UVR over a long usage time. We treated a well‐established hairless C3.Cg/TifBomTac immunocompetent mouse model with two different concentrations of brimonidine (0.33% brimonidine gel or 1% brimonidine cream) and UVR exposure.

## MATERIALS AND METHODS

### Animals

Hairless female C3.Cg/TifBomTac immunocompetent mice (*n* = 125), aged 14–22 weeks at the beginning of the experiment, purchased from Taconic (Ry, Denmark) were used in this study. Mice were anesthetized with 0.05 mL of HypDorm (0.158 mg/mL fentanyl citrate, 5 mg/mL fluanisone, 2.5 mg/mL midazolam), tattooed with consecutive numbers on the abdomen and randomized into five groups. Each group was housed in an individual cage with access to water and a standard diet (Table 1) and maintained at 23–24°C under a 12‐h light‐dark cycle. This study followed recommendations described by national guidelines. All protocols were approved by national (permit number 2014‐15‐0201‐00096) and institutional ethical committees.

**TABLE 1:** Treatment schedule and median number of days until 50% of the mice had a first, second, and third tumor.

Group	Treatment	Irradiation dose (SEDs)	Median days to first tumor (Q_3_–Q_1_)^†^	Median days to second tumor (Q_3_–Q_1_)^†^	Median days to third tumor (Q_3_–Q_1_)^†^
1	1% brimonidine cream before UVR	3	295 (302–288)	302 (309–302)	309 (316–302)
	*P*‐value^‡^		0.000006	0.000004	0.000001
2	0.33% brimonidine gel before UVR	3	267 (295–254)	281 (295–267)	288 (295–274)
	*P*‐value^‡^		0.313	0.447	0.372
3	1% brimonidine cream after UVR	3	274 (281–261)	288 (295–274)	295 (295–288)
	*P*‐value^‡^		0.252	0.172	0.080
4	UVR, no drug treatment (UVR control)	3	267 (274–254)	274 (281–267)	274 (288–267)
5	1% brimonidine cream	NA	No tumor	No tumor	No tumor

SEDs: standard erythema doses; NA: not administered.

^†^ Interquartile range: Q_1_ = 25th percentile and Q_3_ = 75th percentile.

^‡^ The *P*‐value for each group is derived from a comparison with the UVR control group, which had the same UVR dose.

### Drug treatment and light source

An area of skin on the back of each mouse (approximately 15 cm²) was treated for 3 days per week (Monday, Wednesday and Friday) with 25 µL of commercially available 0.33% brimonidine gel (Mirvaso^®^; Galderma R&D, Sophia Antipolis, France) or 25 µL of 1% brimonidine cream for up to 365 days or until death. The 1% brimonidine cream was prepared in‐house. The free form of brimonidine was obtained from Fluorochem (Hadfield, UK). The initial purity of this brimonidine was 95%, and the compound was recrystallized from methanol to increase its purity, as confirmed by ^1^H‐nuclear magnetic resonance spectroscopy and thin‐layer chromatography. Brimonidine was mixed with Unguventum M Cream (Almirall, Reinbek, Germany). One group of mice was irradiated without any drug treatment (the UVR control group), and one group of mice was treated with 1% brimonidine but not UVR. These groups of mice were also followed for 365 days (Table 1). UVR‐treated mice were irradiated with three standard erythema doses (SEDs) of UVR three times per week immediately before or 3–4 h after topical application of 0.33% brimonidine gel or 1% brimonidine cream. The mice were primed with a lower UVR dose during the first 8 weeks of irradiation. The light source used and UVR dose measurement was previously described by Lerche *et al*. (13).

### Study design

A randomization procedure divided the animals into five groups, each consisting of 25 mice (Table 1). Briefly, 1% brimonidine cream (Group 1) or 0.33% brimonidine gel (Group 2) were topically applied, and 3–4 h later, the mice were irradiated with 3× SED UVR. Mice in Group 3 had 1% brimonidine cream topically applied immediately after 3× SED UVR. In the UVR control group (Group 4), animals were left untreated except for UVR. Finally, the nonirradiated mice in Group 5 were treated only with 1% brimonidine cream. For each animal, the first four tumors with at least 1 mm were recorded and followed until three tumors reached 4 mm or one tumor reached 12 mm in diameter (14, 15, 16). Each individual mouse was inspected for tumors once per week. The “time to the first tumor” was defined as the number of days until the appearance of the first 1‐mm‐diameter tumor that after evolved to 4 mm in diameter (17). As a secondary endpoint, the times to the second and third tumors were also recorded as described above. The mice were euthanized after 365 days, or after developing three tumors of 4 mm or one tumor of 12 mm in diameter. The back skin was removed from all mice fixed in 4% buffered formaldehyde. Histopathology was performed on two randomly selected mice from each group to confirm that the tumors were caused by SCC.

### Statistics

Tumor data (i.e. times to the first, second and third tumors) were presented in Kaplan–Meier plots and the groups were compared using the Mantel–Cox log‐rank test. Differences in survival times were considered significant when the *P*‐values were <0.05. All analyses were carried out using SPSS^®^ version 22 for Windows (SPSS Inc., Chicago, IL, USA).

## RESULTS

**Figure 1:**
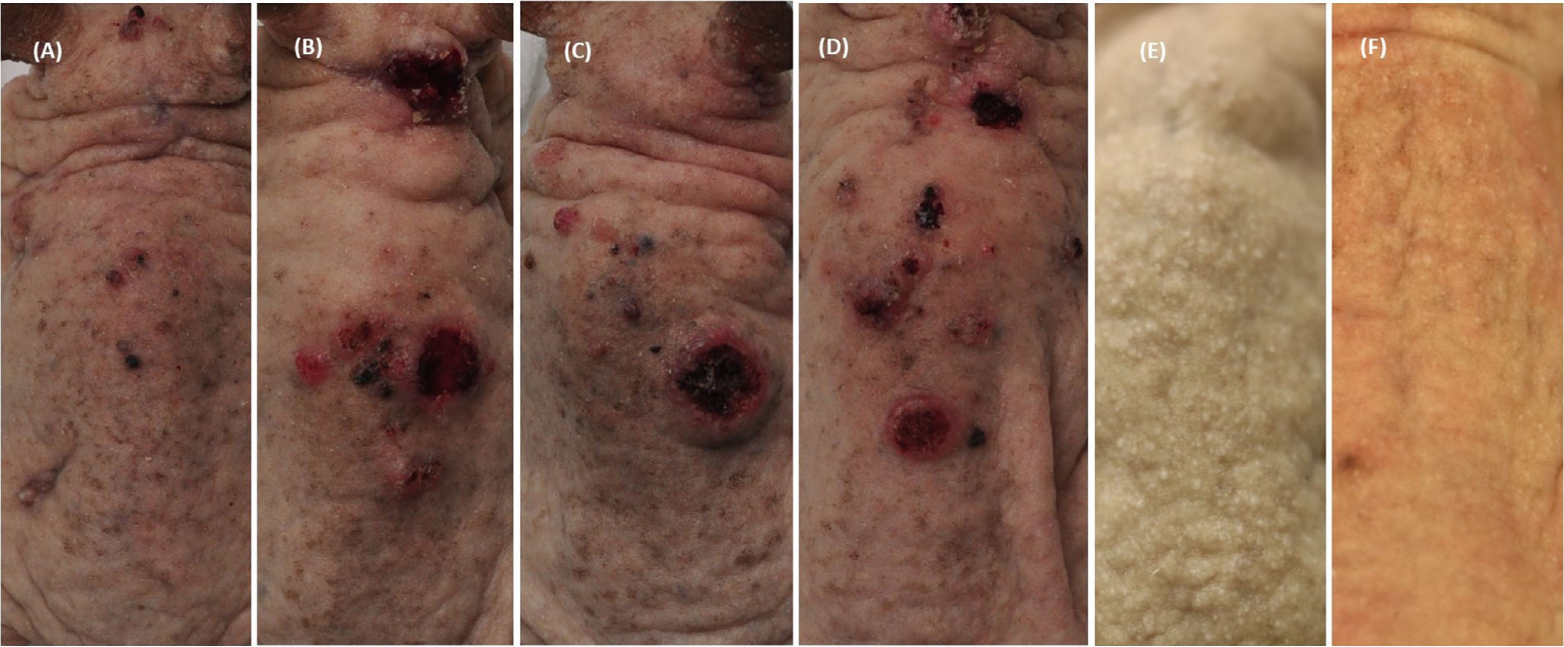
Representative pictures of a mouse from each group. (A) Group 1: 1% brimonidine cream before UVR. (B) Group 2: 0.33% brimonidine gel before UVR. C) Group 3: 1% brimonidine cream after UVR. (D) Group 4: UVR, no drug treatment (UVR control). (E) Group 5: 1% brimonidine cream without UVR. (F) The mice from Group 1 and 5 developed erythema after the application of 1% brimonidine cream.

**Figure 2:**
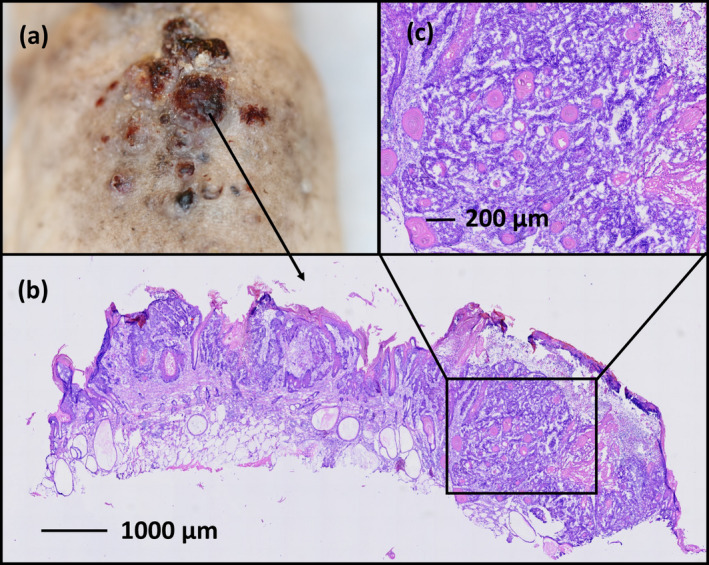
(a) Representative SCC on the back of a UVR control mouse. (b) Overview histology picture (H&E staining) (c). Close up of SCC keratin pearls.

**Figure 3:**
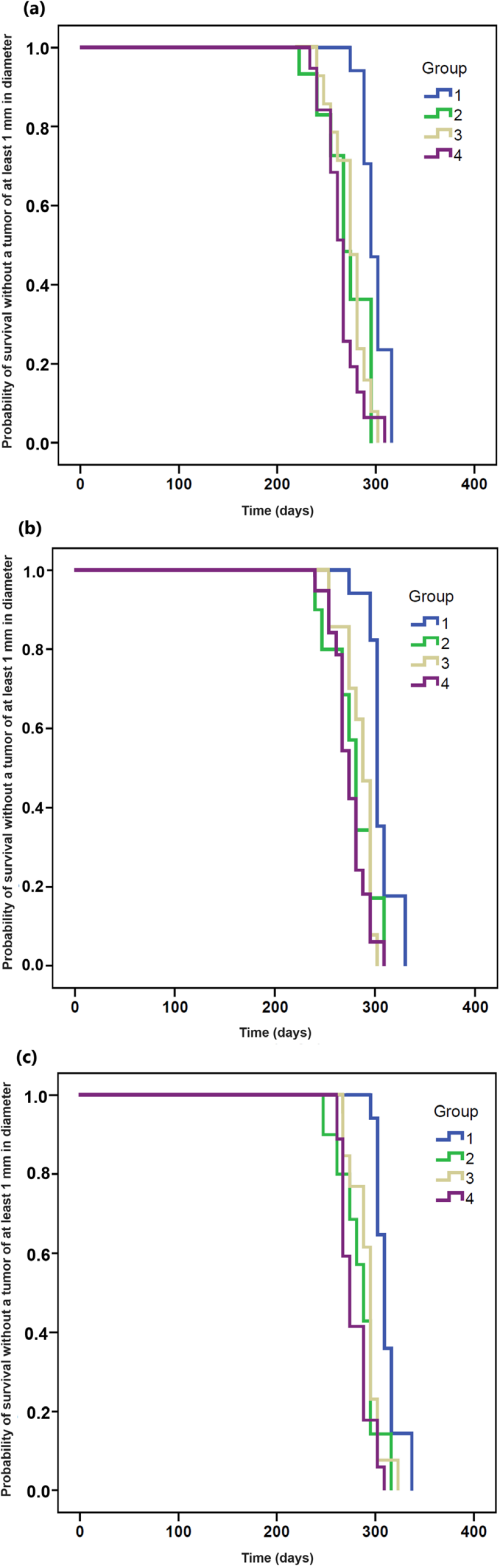
Kaplan–Meier plot showing the probability of survival without a tumor of at least 1 mm in diameter for the groups treated with 1% brimonidine cream before UVR (Group 1), 0.33% brimonidine gel before UVR (Group 2), 1% brimonidine cream after UVR (Group 3) and UVR alone (Group 4). No tumors developed in the nonirradiated Group 5. Panels: (a) first tumor; (b) second tumor; and (c) third tumor.

All UVR‐treated groups of mice developed tumors (Fig. 1A–D). The SCC diagnosis was confirmed in all evaluated tumors (Fig. 2). On the contrary, the nonirradiated 1% brimonidine group (Group 5) did not develop any tumors throughout the 365‐day period of monitoring. Mice treated with 1% brimonidine cream before UVR exposure (Group 1) took a significantly longer time to develop their first tumors than mice in the UVR control group (295 vs. 267 days, *P* = 0.000006; Fig. 3 and Table 1). In Group 2 mice that received 0.33% brimonidine gel before UVR and in Group 4 mice (the UVR control group), the first tumors developed after a median of 267 days (Fig. 3 and Table 1). In addition, there was no significant difference between the median times taken for the first tumors to develop in Group 3 mice, wherein 1% brimonidine cream was applied immediately after receiving UVR treatment, and UVR‐control Group 4 mice (274 vs. 267 days, *P* = 0.252; Fig. 3 and Table 1).

Similar results were observed among the groups for the median times to the second and third tumors. The mice in Group 1, which were treated with 1% brimonidine cream before UVR exposure, took a significantly longer time to develop their second and third tumors than did mice in the UVR control group (302 vs. 274, *P* = 0.000004 and 309 vs. 274, *P* = 0.000001, respectively). There were no significant differences between the median times to tumor development for mice in either Groups 2 or 3 and mice in the UVR control group (all P ˃ 0.05; Fig. 3 and Table 1). Erythema was observed in all mice treated with topical 1% brimonidine approximately 30 min after application (Fig. 1E,F). No weight difference was observed among the groups.

## DISCUSSION

We investigated whether topical brimonidine could delay the development of SCC. The hairless mouse model is the standard tool for evaluating the photocarcinogenicity of drugs and has been widely used (12, 16, 17, 18, 19, 20). We showed that topical 1% brimonidine cream applied before UVR exposure delayed SCC development in hairless mice. In contrast, applying brimonidine after UVR exposure did not significantly delay tumor development compared with mice in a UVR‐only treated control group. Therefore, the delay in SCC development observed in the mice administered 1% topical brimonidine cream before UVR exposure was most likely caused by a UVR absorption by the drug. Brimonidine can absorb light in the UVB wavelength range (280–315 nm), as shown by Bouvier *et al*. (12). However, there can be multiple reasons for a delay in photocarcinogenesis.

When 1% brimonidine was administered to the skin of hairless mice before UVR exposure (Group 1), the drug delayed the development of the first, second and third tumors relative to mice that received UVR alone (Group 4). The application of brimonidine three times per week, 3–4 h before UVR exposure and for 365 days mimics the routine use by patients of a cream or gel in the morning and later exposure to the sun during the day (20). Our results showed a significant difference in the timing of tumor development between mice in Groups 1 and 4, indicating that prior application of brimonidine may protect against UVR exposure and confirming previous results in hairless mice, which showed that brimonidine did not aggravate photocarcinogenesis (12).

However, when a lower concentration of brimonidine was applied before UVR, there was no delay in tumor growth (i.e. no significant difference in the timing of tumor development between mice in Groups 2 and 4). This result agrees with the data presented by Bouvier *et al*. (12), who concluded that initial tumor growth was dose‐dependent. These researchers showed that brimonidine was ineffective below a particular concentration (0.18%), suggesting that the minimal active concentration may be 0.18–1% (12). No vehicle controls groups were included in our study, which is a limitation of the study. However, we have previously investigated the same vehicle used in groups 1, 3 and 5 and it did not alter the time to tumor development or induce erythema in the same strain of mice. However, Bouvier *et al*. (12) demonstrated that the commercial gel‐formulation vehicle alone did not influence the carcinogenic response compared to UVR treatment in the absence of gel. Other studies, such as that by Fowler *et al*. (6), evaluated the optimal concentration, efficacy and safety of brimonidine gel for treating erythema in patients with rosacea and showed that the vehicle formulation did not aggravate erythema or other diseases. The vehicle of the in‐house cream formulation (without titanium dioxide) had no effect on the carcinogenic response (C. M. Lerche, unpublished data). This information supports our finding that 1% brimonidine cream has a protective effect against UVR.

Our study found a statistically significant increase in the length of time taken for a first tumor to develop when mice were treated with brimonidine before instead of after UVR exposure, suggesting UVR absorption by brimonidine. The protective properties of 2% brimonidine were previously linked to a pharmacological effect because in a UVB‐induced epidermal hyperplasia and cell proliferation model, mice treated with 2% brimonidine before or after UVB exposure exhibited similar effects on epidermal hyperplasia and cell proliferation (12). However, the effect on epithelial hyperplasia was low compared to that observed in an endothelial growth factor inhibitor control group of hairless mice, and 0.2% brimonidine had only a small effect (12). Consequently, whether brimonidine could be acting as a sunscreen remained unclear. It was beyond the scope of this paper to investigate the reason for the delay in photocarcinogenesis. However, it could be due to inhibition of DNA damage, oxidative stress, inflammation, immunosuppression and dysregulated signal transduction (21). After our study was ended, we investigated the possibility to estimate the Sun Protection Factor of the brimonidine formulations used in this study to investigate whether there was absorption of UVR by brimonidine. Unfortunately, it was impossible to order more of the free form of brimonidine from Fluorochem (Hadfield, UK). The Sun Protection Factor of the brimonidine formulations could be interesting to evaluate in a future study.

The erythema observed in all mice after 1% brimonidine was applied (Fig. 1) was unexpected. Brimonidine can stimulate vasoconstriction, decrease UVR‐induced erythema (12) and decrease facial erythema in patients with rosacea (5, 6, 22, 23, 24, 25). However, worsening of the erythema after brimonidine use has been reported as an adverse event in clinical trials (23, 25, 26, 27) and has been described as paradoxical erythema (28). Proposed physiological mechanisms are local inflammation and a higher concentration of brimonidine in the skin, leading to a "spillover" effect to other receptor subtypes resulting in vasodilation and/or genetic predisposition (28). In our study, the underlying mechanism for observed erythema in mice treated with 1% brimonidine is not clarified, but the erythema was persistent throughout the entire study and not observed in the group treated with 0.33% brimonidine gel. The murine skin is thinner than human skin, so a higher concentration of brimonidine within the skin leads to a saturation of receptors, and a "spillover" effect to other subtype receptors could explain the observed erythema. It could be prevented in future studies using a thinner layer of cream or a gel with a lower concentration of brimonidine.

Erythema is caused by vascular and inflammatory events (28) and has been associated with an increased risk of carcinogenesis (29, 30). However, erythema was not associated with an increased risk of carcinogenesis here because a protective effect was observed. A few studies have investigated the association between skin cancer and rosacea. A study with 140 patients did not find an increased occurrence of skin cancers compared to age‐ and sex‐matched controls (31). In contrast, a large register study by Egeberg *et al*. (32) from Denmark with 49,475 patients with rosacea and 4,312,213 subjects from the general population showed an increased risk of keratinocyte cancer in patients with rosacea (hazard ratio; 1.36 and 95% confidence interval; 1.26–1.47). Also, Li *et al*. (33) reported an increased relative risk of BCC among 6015 female nurses with rosacea (RR 1.50; 95% CI 1.35–1.67). The increased risk is not clarified but could be due to higher UV exposure, inflammation, or maybe medical treatments for rosacea (33).

Most importantly, our results show that topically administered brimonidine did not accelerate photocarcinogenesis or induce dermal carcinogenicity in hairless mice. Furthermore, topical 1% brimonidine cream applied before UVR exposure delayed SCC development in hairless mice. This protective effect is probably due to UVR absorption because no inhibition of photocarcinogenesis was observed in mice treated with 1% brimonidine cream after UVR exposure but could be due to several reasons.
